# Research on distribution network fault processing technology based on knowledge of graph

**DOI:** 10.1371/journal.pone.0295421

**Published:** 2023-12-14

**Authors:** Qiang Li, Feng Zhao, Li Zhuang, Jiangwen Su, Xiaodong Zhang

**Affiliations:** 1 State Grid Information & Telecommunication Co., Ltd., Beijing, China; 2 FuJian YiRong Information Technology Co.Ltd, Fujian Fuzhou, China; TU Wien: Technische Universitat Wien, AUSTRIA

## Abstract

Safety and reliability are the basis of the development of a distribution network. To analyze the risk transmission process in the distribution network and ensure the safe and reliable operation of the power system, this paper intends to use the knowledge graph method to realize the risk analysis of the distribution network information system. Firstly, the knowledge graph method is used to extract and integrate the risk knowledge of the multi-dimensional information collected by the distribution network. Secondly, the knowledge graph model of distribution network risk analysis is constructed, and the multi-dimensional distribution network fault handling and knowledge graph construction oriented to the feeder and platform area are designed. The distribution line parameters of the low-voltage distribution network model, neutral point grounding mode, and different fault types are analyzed, and the non-planned island is searched based on the knowledge graph adjacency matrix. Finally, combined with the simulation experiment, it is verified that the proposed method can effectively depict the information risk process of the distribution network. The structure of this paper starts from the multi-node complex distribution network, combined with a knowledge graph and deep learning method, which can solve the distribution network fault more quickly.

## 1. Introduction

At present, with the deepening of the physical integration of power information, the distribution system presents the characteristics of a complex structure and multiple external risk factors, and the fault forms of the distribution network are increasingly complex. The existing power grid regulation system focuses on collection, monitoring, and analysis, and has accumulated a large number of multi-source heterogeneous text data [1–6], but the current distribution network fault disposal still mainly depends on the subjective decision of the regulator.

In addition, the existing text data of the distribution network presents the characteristics of multiple types and small volumes, which makes it difficult to effectively support the training of the conventional deep learning model [7–12]. Therefore, it is urgent to extract and refine the multi-source heterogeneous distribution network text data into knowledge with the help of intelligent technology and organize this knowledge into structured and visual representation forms. Related contents of multi-dimensional distribution fault outage risk situation awareness, such as fault monitoring, non-contact detection, fault warning, etc., construct knowledge graphs of various measurement states, topologies, data, etc., and establish corresponding relationships [13–19]. Knowledge graph technology is mainly used to enhance search services, and its application in power systems is still in its infancy. The main application scenarios include heterogeneous data management of power systems, power grid fault-assisted decision-making, and power equipment health management.

In this study, heterogeneous text data of the distribution network is considered and different knowledge extraction methods are designed. The construction of a distribution network fault disposal knowledge map requires natural language processing for unstructured text data, and the knowledge map method is used to realize the risk analysis of the distribution network information system. The distribution network first uses the knowledge graph method to extract and integrate the risk knowledge of the multi-dimensional information collected by the distribution network and realizes the static analysis of the system risk of the distribution network. Secondly, the knowledge graph model of distribution network risk analysis is constructed, and the multi-dimensional distribution network fault disposal and knowledge graph oriented to the feeder and station area are designed. The knowledge graph model of distribution network risk analysis is constructed, and the multi-dimensional distribution network fault disposal and knowledge graph oriented to the feeder and station area are designed. The distribution line parameters and neutral point grounding mode of the low-voltage distribution network model are considered. Different fault types are analyzed and non-planned islands are searched based on the knowledge graph adjacency matrix. Finally, combined with the simulation experiment, it is verified that the proposed method can effectively depict the information risk process of the distribution network. It is verified that the proposed method can effectively describe the information risk process of the distribution network.

The knowledge graph itself is an intelligent algorithm. At present, a map of the guaranteed power supply site is drawn by CAD software, which is incompatible, not general, and not friendly in the system, and data cannot be correlated. Therefore, this paper intends to apply this intelligent algorithm of knowledge graph to analyze the distribution network system.

At the same time, based on the built knowledge database, through knowledge reasoning and deep learning technology, and combined with the real-time operating status data of equipment in the power grid management platform, the power supply path can adjust the direction and focus of the power supply according to the power grid operation mode in time, and achieve penetrating management. Through rapid and intelligent risk fault sensing technology, automatic discovery and real-time perception of risk points in the path of guaranteed power supply are assisted, which improves the quality and efficiency of risk management and makes the service management of guaranteed power supply more lean and digital.

Since the occurrence of distribution network faults is accompanied by many accidental and inevitable factors [3–6, 20–22], which are affected by many factors (weather, temperature, line type, etc.), identification of distribution network fault causes based on multi-source information fusion will effectively improve the identification accuracy. Nowadays, machine learning is developing rapidly, such as logistic regression algorithms, neural network algorithms, and support vector machine algorithms. However, the feature extraction ability of these methods still has some shortcomings, such as not easily obtaining the global optimal solution, resulting in poor generalization power and low recognition accuracy. In literature [9, 23–25], the non-convex model is transformed into a linear model through dimensionalization, relaxation, or data-driven methods, and then solved with mature solvers. Literature [26] proposes a distributed fault location method based on the Bayesian model, which has good effects on the adaptability and fault tolerance of active distribution networks. However, this method relies on distributed communication and calculation and requires prior probability in Bayesian inference to be obtained in advance. However, the knowledge graph method has better feature extraction and sample learning ability. Combined with input sample data with more dimensions, it provides a new means for multi-source information fusion to realize fault-cause identification of the distribution network. According to the data characteristics and development trend of the electric Internet of Things, literature [27] proposes a framework of knowledge graph in the electric power field based on the idea of an extended domain knowledge graph spectrum and expounds the technical route and implementation scheme of the practical application of knowledge graph in the electric power field in combination with specific application scenarios such as power grid dispatching fault processing, power transport inspection work order processing, and power customer service intelligent question and answer. Literature [28] proposes a fault diagnosis method based on a knowledge graph, realizes entity extraction with a combination model, and uses knowledge graph construction technology to construct and apply knowledge graphs for power grid fault disposal. Literature [29] proposed the construction framework and method of knowledge graph of distribution network fault disposal and used a pre-training method to build a deep learning model, to realize named entity identification of fault disposal data. Finally, the knowledge graph was stored and managed with the graph database Neo4j.

This paper adopts the intelligent algorithm of deep learning of neural networks. Knowledge graphs can find faults in distribution networks faster, and make a more accurate judgment on future faults through deep learning.

The innovation of this paper mainly lies in.

The knowledge graph model of distribution network risk analysis is constructed, and the construction of a multi-dimensional distribution network fault disposal and knowledge graph oriented to feeders and stations is designed. The knowledge graph model of distribution network risk analysis is constructed, and the construction of a multi-dimensional distribution network fault disposal and knowledge graph oriented to feeders and stations is designed.

Considering the distribution line parameters, neutral grounding mode, and different fault types of the low-voltage distribution network model, the search for a non-planned island based on the knowledge graph adjacency matrix is studied.

Combined with simulation experiments, it is verified that the proposed method can effectively depict the information risk process of the distribution network. It is verified that the proposed method can effectively describe the information risk process of the distribution network.

## 2. Analysis of knowledge graph construction technology

The power grid stability situation knowledge map uses the graph model to describe the relationship between power grid stability situation knowledge and power grid physical equipment and realizes the direct expression of power grid stability situation knowledge [10–15]. The power grid stability situation knowledge graph can be divided into a system-level knowledge graph and a device-level knowledge graph according to the dispatcher’s concern level, which realizes the hierarchical knowledge of the power grid stability situation and helps dispatchers intuitively understand the power grid stability situation information [16–19, 30].

The system-level knowledge map mainly describes the system knowledge of the power grid, including the physical topology information between devices, the overall state information of the system, various stability indicators of the system, the fault/disturbance events occurring in the system, and the corresponding regulation strategies of the fault/disturbance events. Through the system-level knowledge graph, the scheduler can direct.

**Fig 1:**
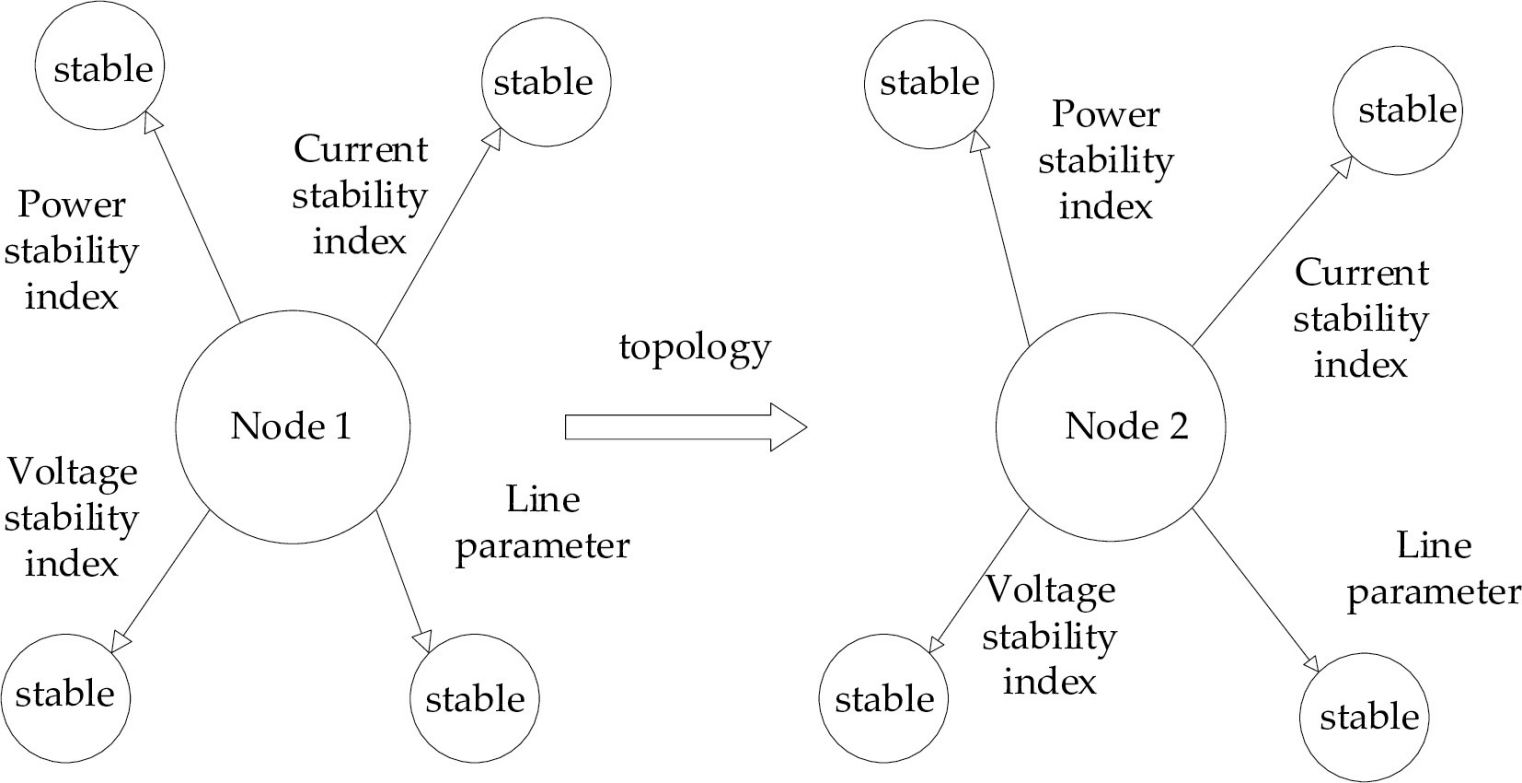
Shows the grid stability situation knowledge map usage graph model.

Observe and understand the overall operation state of the power grid. When the scheduler wants to focus on a device or all devices in a plant, he can access the device-level knowledge graph. The device-level knowledge graph describes device knowledge, including physical topology information between devices, real-time device measurement information, device static model parameters, and device indicators. In addition, the equipment level knowledge graph of the power grid stability situation also introduces the knowledge of equipment production and operation and integrates the information of equipment manufacturer, model, service life, and maintenance record into the equipment level knowledge graph. The weight is divided according to the attention of dispatchers, so that the content of the equipment level knowledge graph is richer and more comprehensive, and helps dispatchers fully grasp the operation status of equipment. Fig 1 shows the grid stability situation knowledge map usage graph model.

The nodes in the specified grid are the transformer area of the distribution network, The digraphs used in this paper are directed graphs.

## 3. Construction of low-voltage distribution network model

### 3.1 Introduction and parameters of power lines in the distribution network

The distribution network mainly consists of distribution lines, distribution primary equipment, and distribution terminal equipment. When a fault occurs in the distribution network, the electrical characteristics of transmission lines with different lengths and structures are also different. To ensure the stability of the power network and the safety of the power supply, a series of parameters such as fault setting, and steady-state transient characteristics must be calculated accurately. Distribution lines have three characteristics: resistance, sensitivity, and capacity. Characteristics are resistance, inductance, and capacitance. Multiple lumped parameters can be used to simulate the equivalent simulation of transmission lines in various cases.

The power line resistance of the distribution network can be calculated by the following formula:

$$r_{1}=\frac{\rho }{s},\;R_{l}=r_{1}•l$$ (1)

*r*_1_ Represents the resistance per unit length, *R*_*l*_ represents the resistance of the wire, *ρ* represents the resistivity of the material of the wire, *s* represents the cross-sectional area of the wire, *l* and represents the length of the line, because conductor resistance varies with temperature. So you need a temperature correction.


$$r_{t}=r_{20}[1+\alpha (t-20)]$$ (2)

**Fig 2:**
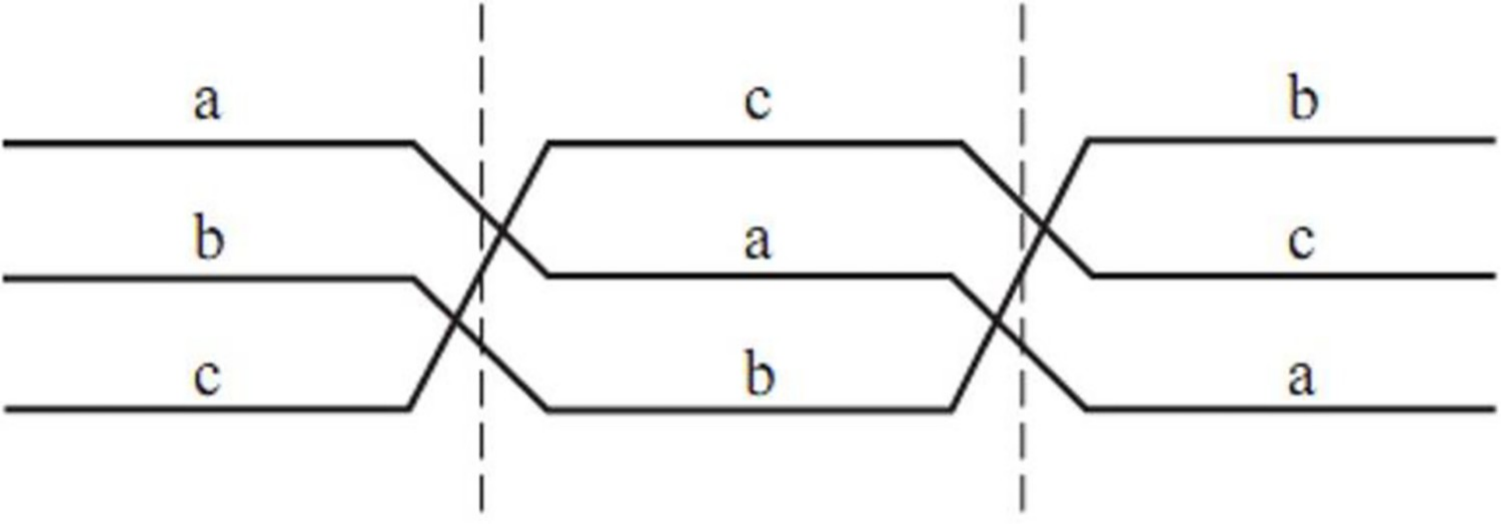
Overhead line transposition.

The magnetic field effect of power lines in the distribution network is represented by reactance. To increase the transmission capacity and equalize the reactance parameters of the lines, the entire cycle conversion should be performed. Fig 2 Overhead line transposition.


$$x_{1}=2\pi f(4.61l_{g}\frac{Dm}{r}+0.5\mu_{r})\times 10^{-4}=0.1445l_{g}\frac{Dm}{r}+0.0157$$ (3)

*r* Represents the calculated radius of the wire, *f* represents the frequency, *Dm* represents the geometric mean distance of the three-phase wire, the distance between the three-phase wire AB, BC, CA, *D*_*ab*_, *D*_*bc*_, *D*_*ca*_ and the geometric mean distance is solved by Formula (4).


$$Dm=\sqrt[{3}]{D_{ab}D_{bc}D_{ca}}$$ (4)

**Fig 3:**
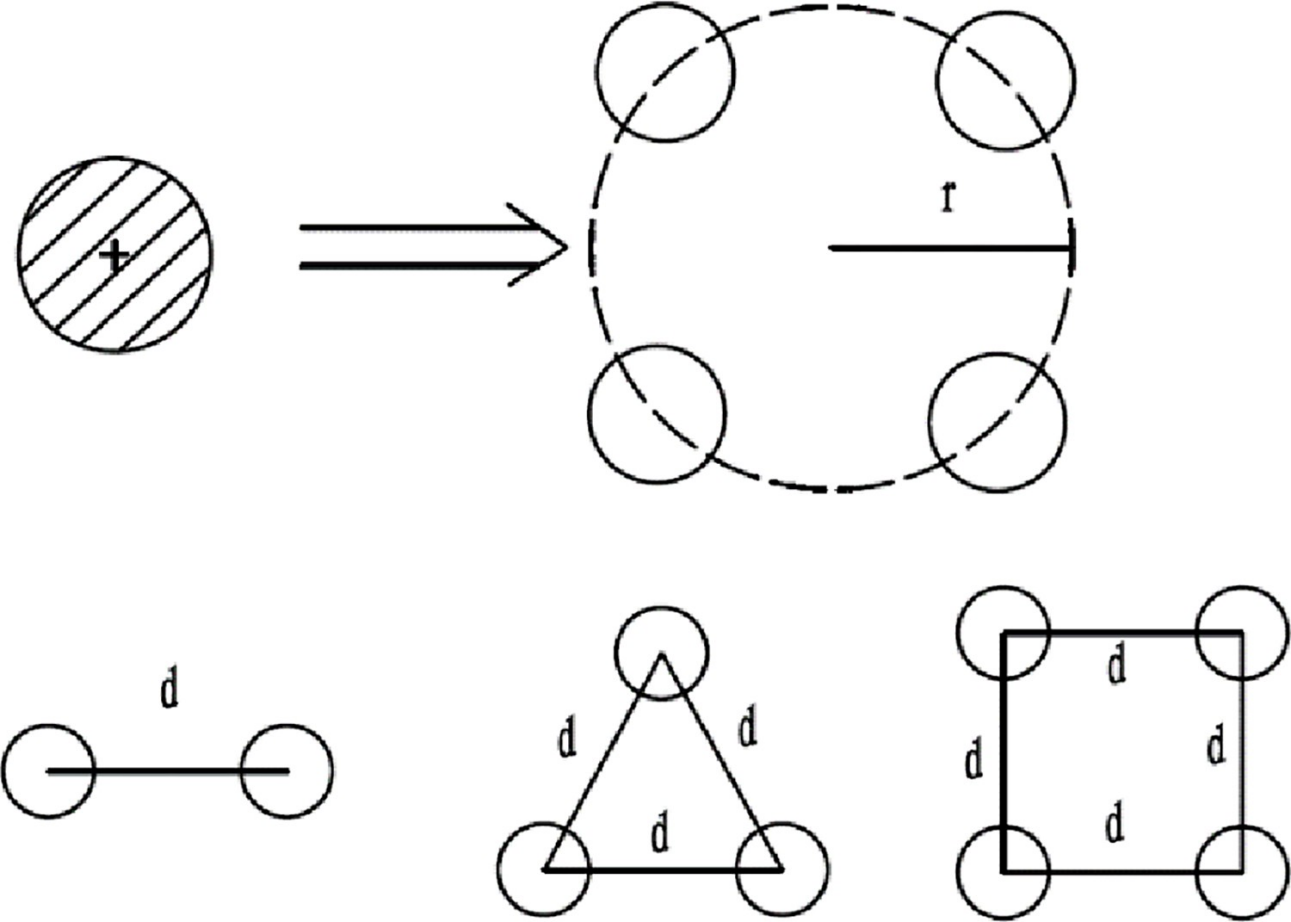
Schematic diagram of a single-line split conductor.

If each phase line is divided into n lines, it reduces the reactance per unit length. Fig 3 Schematic diagram of single line split conductor.


$$x_{1}=0.1445l_{g}\frac{Dm}{r_{eg}}+\frac{0.0157}{n}$$ (5)

The critical corona generation voltage is

$$V_{CR}=49.3m_{1}m_{2}\delta \mathrm{lg}\frac{D}{r}$$ (6)

*m*_1_ Represents the roughness coefficient of the wire surface, *m*_2_ represents the coefficient considering the influence of weather, *δ* represents the air density, *D* represents the distance between phases, *r* represents the calculated radius of wire.

In practical application, the corona is generally not allowed under normal weather conditions. According to Eq (6), parameters related to corona, and critical voltage include wire phase spacing and wire calculation radius. Changes in these two parameters do not significantly change the corona critical voltage. So you can ignore the conductivity in the general design.

### 3.2 Steady-state characteristics analysis of single-phase grounding in the ungrounded neutral system of distribution network

Unlike the neutral grounding system described above, the neutral grounding system through resistance has no more common applications than the two described above. Neutral resistance grounding means that the neutral point of the system is grounded through appropriate resistance. This reduces the potential rise near the point of failure of the system. It improves personal safety and equipment safety. The adaptability of the system is poor and the application range is very limited. If the loop network power supply is not realized, frequent turns will deteriorate the working environment and accelerate the aging of circuit breakers. Neutral resistance grounding systems are usually used in power distribution networks where the earthed capacitance current is less than 10A.

Line impedance and grounding parameters are very important parameters in a power system. Line type, length and fault degree of the line will have a key impact on simulation calculation and actual engineering calculation

Transient analysis of single-phase grounding fault.

**Fig 4:**
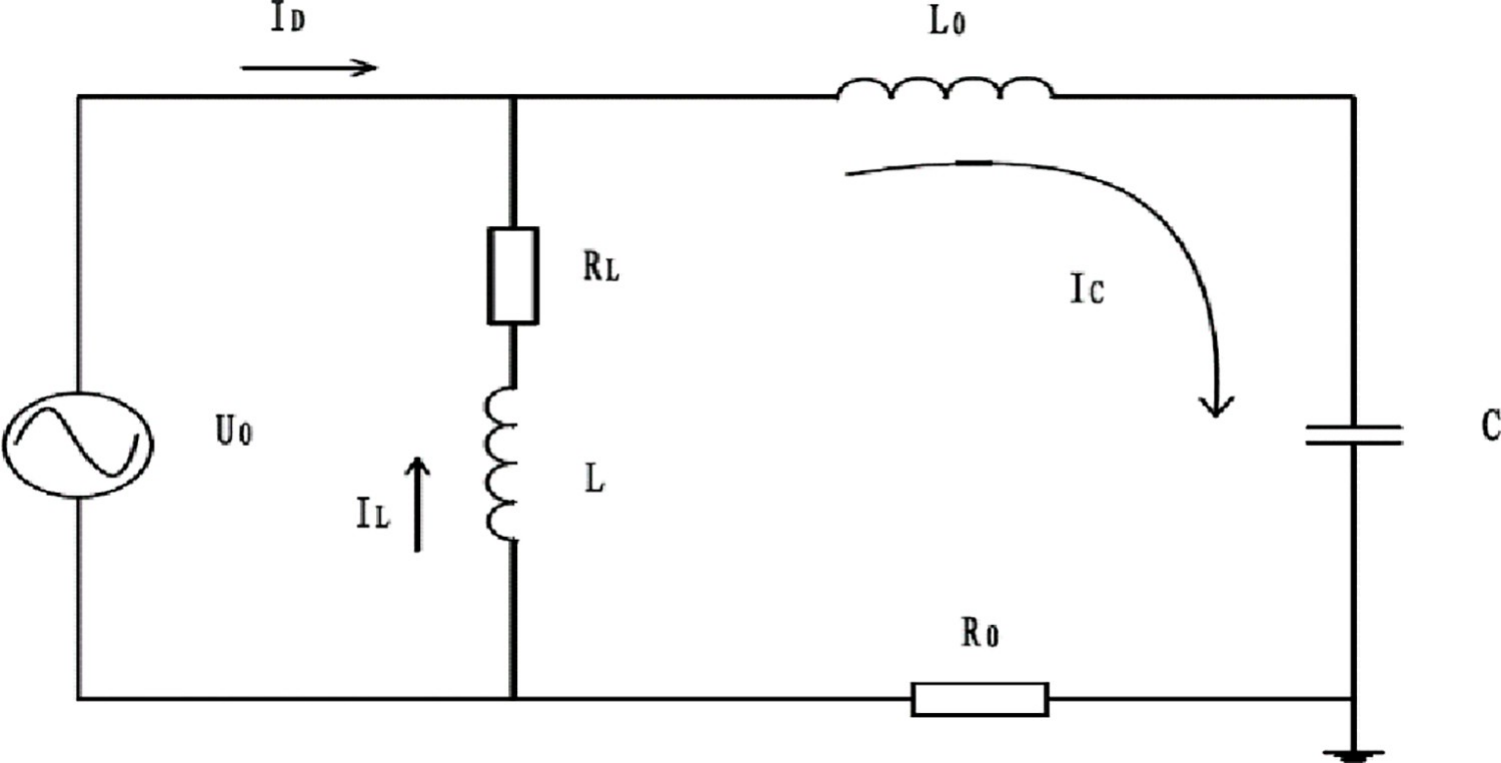
Transient equivalent circuit when single-phase grounding. *U*_0_ Represents an equivalent zero-sequence voltage source. *I*_*D*_ Represents the transient ground current. *R*_*L*_ Represents active power loss resistance. *L* Represents the inductance of the arc suppression coil. *I*_*L*_ Represents transient inductive current. *L*_0_ Represents the inductance of a three-phase circuit in a zero-sequence loop. *R*_0_ Represents the equivalent resistance of the zero sequence loop. *C* Represents capacitance to the ground. *I*_*C*_ Represents transient capacitance current.

The system is generally in a steady state process. The transient equivalent circuit of single-phase grounding is shown in Fig 4.

(1) transient capacitance current

During the transient ground fault, the system vibrates strongly. The inductance value of the line is much less than that of the arc suppression coil. Therefore, it can be considered that the branch formed in series is disconnected and the inductive current is ignored. Let me write down the equation;

$$R_{0}\geq 2\sqrt{\frac{L_{0}}{C}}$$ (7)

At that time $R_{0}\geq 2\sqrt{\frac{L_{0}}{C}}$, the resistance was high, and it reached a steady state very quickly. At that time $R_{0}\geq 2\sqrt{\frac{L_{0}}{C}}$, the transient process of periodic oscillation.

The transient capacitance current after the fault is

$$I_{C}=I_{C}^{\prime }+I_{C}^{\prime \prime },$$ (8)

$$I_{C}^{\prime }+I_{C}^{\prime \prime }=0,I_{Cm}=U_{Cm}wC$$ (9)

$$I_{C}=I_{C}^{\prime }+I_{C}^{\prime \prime }=I_{Cm}(\frac{\omega_{f}}{\omega }\sin\varphi \sin\omega_{f}t-\cos\varphi \cos\omega_{f}t)e^{-\delta t}+I_{Cm}\cos(\omega t+\varphi )$$ (10)

:

Transient inductance current is shown in the following equation

$$U_{m}\sin(wt+\varphi )=I_{L}R_{L}+N\frac{d\phi_{L}}{dt}$$ (11)

Expressions that can be listed

The ground current in the transient process is:

$$I_{L}=I_{Lm}[\cos\varphi e^{-\frac{t}{\tau_{L}}}-\cos(wt+\varphi )]$$ (12)

The ground current in the transient process is:

$$I_{D}=I_{C}+I_{L}=(I_{Cm}+I_{Lm})\cos(wt+\varphi )+(\frac{\omega_{f}}{\omega }\sin\varphi \sin\omega_{f}t-\cos\varphi \cos\omega_{f}t)e^{-\delta t}+I_{Lm}\cos\varphi e^{-\frac{t}{\tau_{L}}}$$ (13)

What is expressed in the formula is the steady-state component of the ground current, while the components of the time-ground transient current expressed in the following items have the biggest influence on the period and magnitude of the ground current. Table 1 is the Current value of ground capacitance added to the substation.

**Table 1:** Current value of ground capacitance added to substation.

**Rated voltage**	8	10	16	35	63	110
**Added value**	18	16	15	13	12	10

Single-phase earthed capacitance current of an overhead line *I*_*CO*_

$$I_{CO}=K_{O}K_{S}U_{N}L_{O}•10^{-3}$$ (14)

Single-phase earthed capacitance current of a cable *I*_*CC*_

$$I_{CC}=0.1U_{N}L_{C}$$ (15)

ground capacitance current of the substation

$$I_{C}=K•(I_{CO}+I_{CC})$$ (16)

The capacity of the arc suppression coil

$$S_{X1}=K_{\alpha }I_{C}\frac{U_{N}}{\sqrt{3}}$$ (17)

The differential equation of arc conductance g describes an arc in the air:

$$\frac{dg}{dt}=\frac{1}{\tau }(G-g)$$ (18)

Fixed arc conductance is defined as:

$$G=\frac{\left|i_{arc}\right|}{u_{st}}$$ (19)

$$u_{st}=u_{0}+r_{0}\cdot |i_{arc}|$$ (20)

$$u_{0}=0.9\frac{kV}{m}\cdot l_{arc}+0.4kV$$ (21)

$$r_{0}=40\frac{m\Omega }{m}\cdot l_{arc}+8m\Omega$$ (22)

The relationship between arc burning time constants can be defined by the following relation:

$$\tau =\tau_{0}\cdot {\left(\frac{l_{arc}}{l_{0}}\right)}^{\alpha }$$ (23)

Because arc length variation is highly dependent on the environment, it is difficult to take these random effects into account accurately in arc models. Therefore, the arc reignition after quenching is not considered in this arc model.

## 4. Construction of knowledge map of distribution network system

According to whether it is pre-planned or not, island running can be divided into planned island running and non-planned island running. According to the capacity of the power supply, the running state before the failure, and the size of the local load, the reasonable island area is determined in advance, and the stable operation of the small system can be guaranteed after being disconnected from the main system, which is called the planned island. In the case of a non-planned island, the distributed power supply runs in conjunction with the adjacent load. Because the matching between the load and the distributed power supply is not considered, the problem of busbar voltage and line load crossing the limit is easy to occur. When different components of the distribution network fail, the distribution network fault set will be formed, and different fault operation schemes will be formed under different distribution network faults, which will affect the power flow distribution, node voltage distribution, and load outage of the distribution network. Therefore, it is necessary to propose the island analysis method.

The distribution network is a network composed of many electrical components connected. Electrical equipment in feeders can be regarded as nodes in graph theory, and the connections between devices as arcs in graph theory to obtain graphs in graph theory. The non-planned island needs to search for the load connected to the power supply after failure. Here, we do not care about the path length between the power supply and the load but only care about the connectivity. We can use the connectivity knowledge in graph theory to search for a planned island. Adjacency matrix and correlation matrix are commonly used to describe graphs in research. The adjacency matrix is a matrix representing adjacency relations between vertices. The correlation matrix uses a matrix to represent the relationship between each point and each edge. In practical applications, it is easy to generate the adjacency matrix from the distribution network, and it is easy to determine whether any two vertices in the graph have edges connected.

1) Adjacency matrix

Let G = (V, E) be a graph with n nodes V = {v1, v2…, vn}

$$A(G)=(\begin{array}{cccc} a_{11} & a_{12} & \cdots & a_{1n}\\ a_{21} & a_{22} & \cdots & a_{2n}\\ \vdots & \vdots & \cdots & \vdots \\ a_{n1} & a_{n2} & \cdots & a_{nn} \end{array})$$ (24)

The adjacency matrix of an undirected graph is symmetric, while the adjacency matrix of a directed graph is not necessarily symmetric.

(2) Reachable matrix.

Let G = (V, E) be a graph with n nodes V = {v1, v2…, vn}. Then the equation of order n is called the reachable matrix of G.

A matrix is used to describe the degree that can be reached between nodes of a directed connection graph after a certain length of the path.


$$P(G)=(\begin{array}{cccc} p_{11} & p_{12} & \cdots & p_{1n}\\ p_{21} & p_{22} & \cdots & p_{2n}\\ \vdots & \vdots & \cdots & \vdots \\ p_{n1} & p_{n2} & \cdots & p_{nn} \end{array})$$ (25)

The accessibility matrix indicates whether there is a path between any two nodes in the graph and whether there is a loop at any node. When discussing reachability, we are interested in whether there is a path from Vi to Vj, not in the length and number of paths.

The accessibility matrix indicates whether there is a path between any two nodes in the graph and whether there is a loop at any node. When discussing reachability, we are interested in whether there is a path from Vi to Vj, not in the length and number of paths.

Search non-planned islands based on the knowledge graph adjacency matrix, the specific calculation procedure is as follows:

① Enter the node adjacency matrix of the network. The branch information of the network is the line number, nodes at both ends, fault edges, and location of the distributed power supply nodes (g1, g2, g3…… gn).
② According to the branch information, the connection relation value at both ends of the faulty edge in the adjacency matrix is changed to 0
③ The reachable matrix is calculated according to the modified adjacency matrix.
④ Power source 1 of node g1 starts to search, extract the data of row g1 of the reachable matrix, search for the element with a value of 1, and the corresponding column number is the node connected with the power source 1, forming the non-planned island. If the node group contains the location nodes of all distributed power supplies (g1, g2, g3…… gn), indicating that all power sources form a non-planned island, then the calculation is complete, or proceed to the next step.
⑤ Exclude the power nodes connected to power 1, and search the remaining power nodes again until all power supplies are searched.

## 5. Example analysis

**Fig 5:**
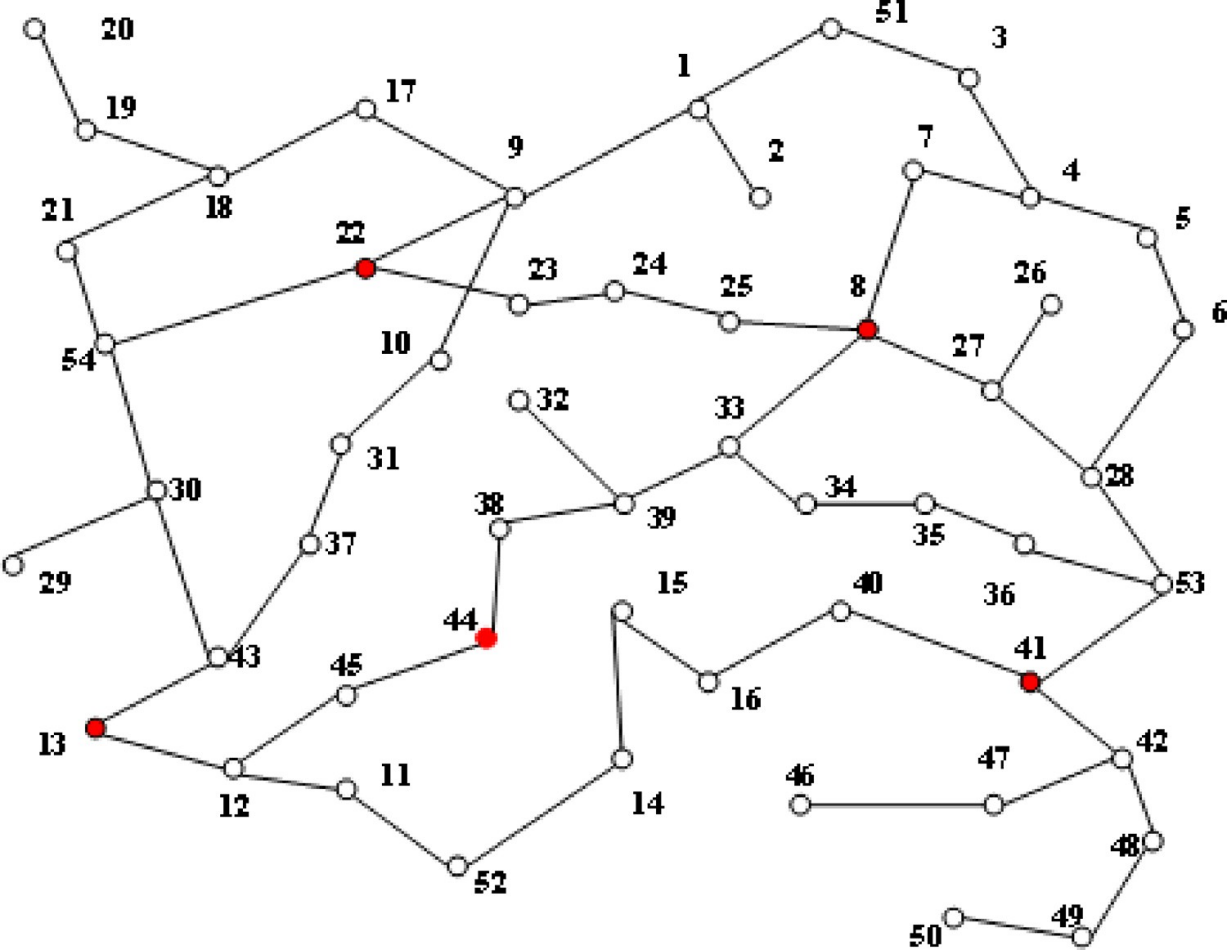
Structure of 54-node distribution network.

The structure diagram of the 4-node distribution network is shown in Fig 5. Node parameters and branch parameters are shown in Tables 2 and 3 respectively.

**Table 2:** Node parameters of the 54-node distribution network.

Node	Active power (kW)	reactive power	Node	Active power (kW)	reactive power
1	3300	330	2	1100	110
3	400	40	4	1400	140
5	2000	200	6	600	60
7	200	20	8	1500	150
9	1900	190	10	2000	200
11	200	20	12	1000	100
13	900	90	14	800	80
15	1000	100	16	1300	130
17	500	50	18	900	90
19	1000	100	20	500	50
21	500	50	22	500	50
23	500	50	24	500	50
25	600	60	28	400	40
29	600	60	30	2000	200
31	600	60	33	1800	180
34	900	90	36	200	20
37	1000	100	39	800	80
40	1000	100	41	300	30
44	500	50	45	500	50
47	500	50	48	500	50
51	2000	200	52	800	80
53	600	60	54	300	30

**Table 3:** Branch parameters of the 54-node distribution network.

Head node	Terminal point	resistance	reactance	Head node	Terminal point	resistance	reactance
51	1	0.3	0.3	27	28	0.22	0.22
51	3	0.28	0.28	6	28	0.4	0.4
3	4	0.24	0.24	54	30	0.22	0.22
4	7	0.26	0.26	30	29	0.28	0.28
4	5	0.28	0.28	30	43	0.37	0.37
7	8	0.26	0.26	43	37	0.2	0.2
5	6	0.21	0.21	37	31	0.18	0.18
1	9	0.34	0.34	31	10	0.2	0.2
1	2	0.28	0.28	13	43	0.24	0.24
9	10	0.6	0.6	12	45	0.2	0.2
52	14	0.4	0.4	45	44	0.2	0.2
14	15	0.3	0.3	44	38	0.24	0.24
15	16	0.26	0.26	38	39	0.39	0.39
52	11	0.2	0.2	39	32	0.32	0.32
11	12	0.28	0.28	39	33	0.2	0.2
12	13	0.34	0.34	33	8	0.18	0.18
19	20	0.22	0.22	33	34	0.2	0.2
18	19	0.2	0.2	34	35	0.2	0.2
17	18	0.3	0.3	35	36	0.2	0.2
9	17	0.4	0.4	53	36	0.24	0.24
18	21	0.26	0.26	53	28	0.3	0.3
54	21	0.22	0.22	53	41	0.32	0.32
54	22	0.32	0.32	41	40	0.22	0.22
9	22	0.4	0.4	40	16	0.22	0.22
22	23	0.31	0.31	41	42	0.3	0.3
23	24	0.2	0.2	42	48	0.2	0.2
24	25	0.2	0.2	48	49	0.3	0.3
25	8	0.2	0.2	49	50	0.2	0.2
8	27	0.31	0.31	42	47	0.26	0.26
27	26	0.24	0.24	47	46	0.26	0.26
				46	14	0.3	0.3

**Fig 6:**
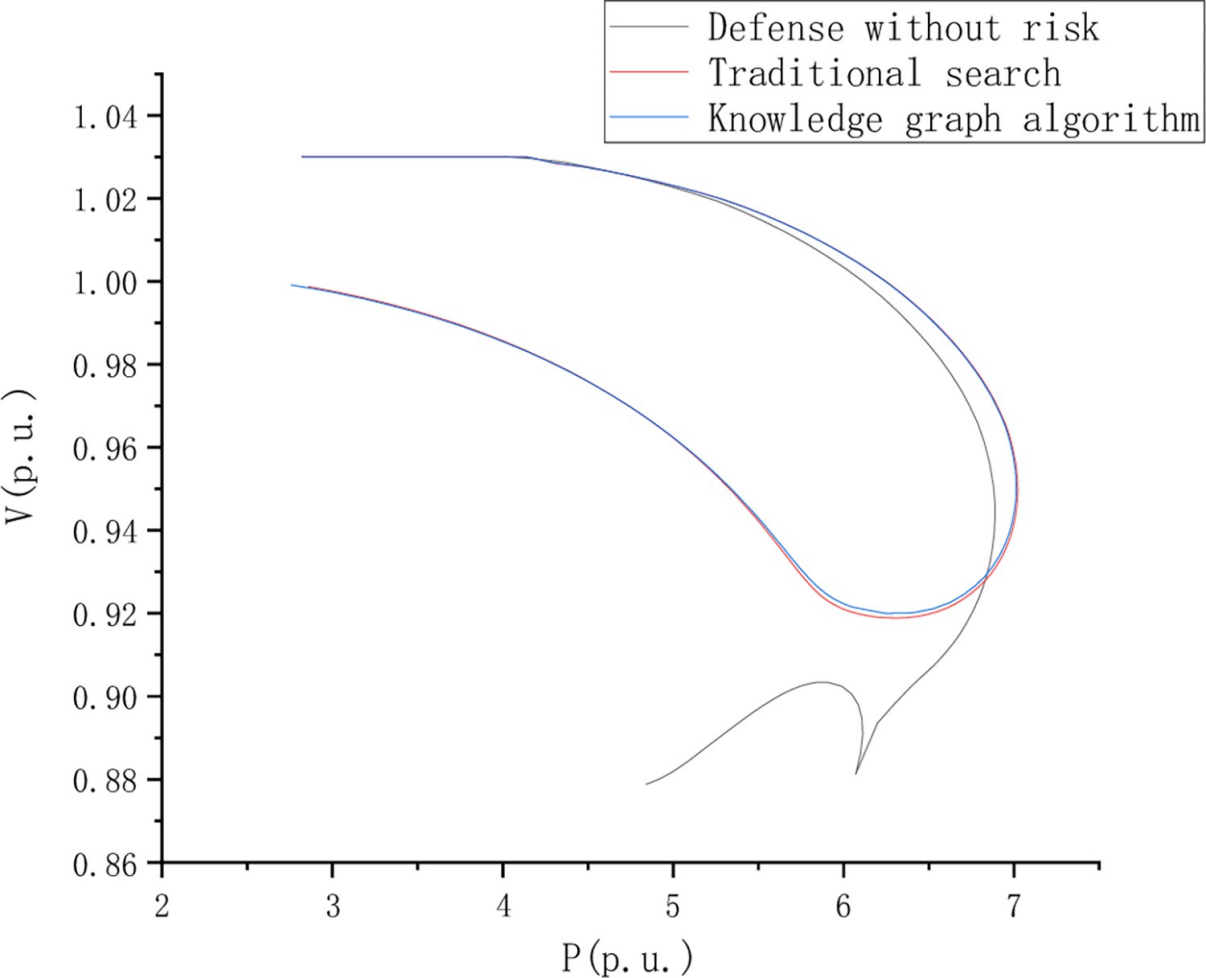
PV curve of node 8 with load shortage.

**Fig 7:**
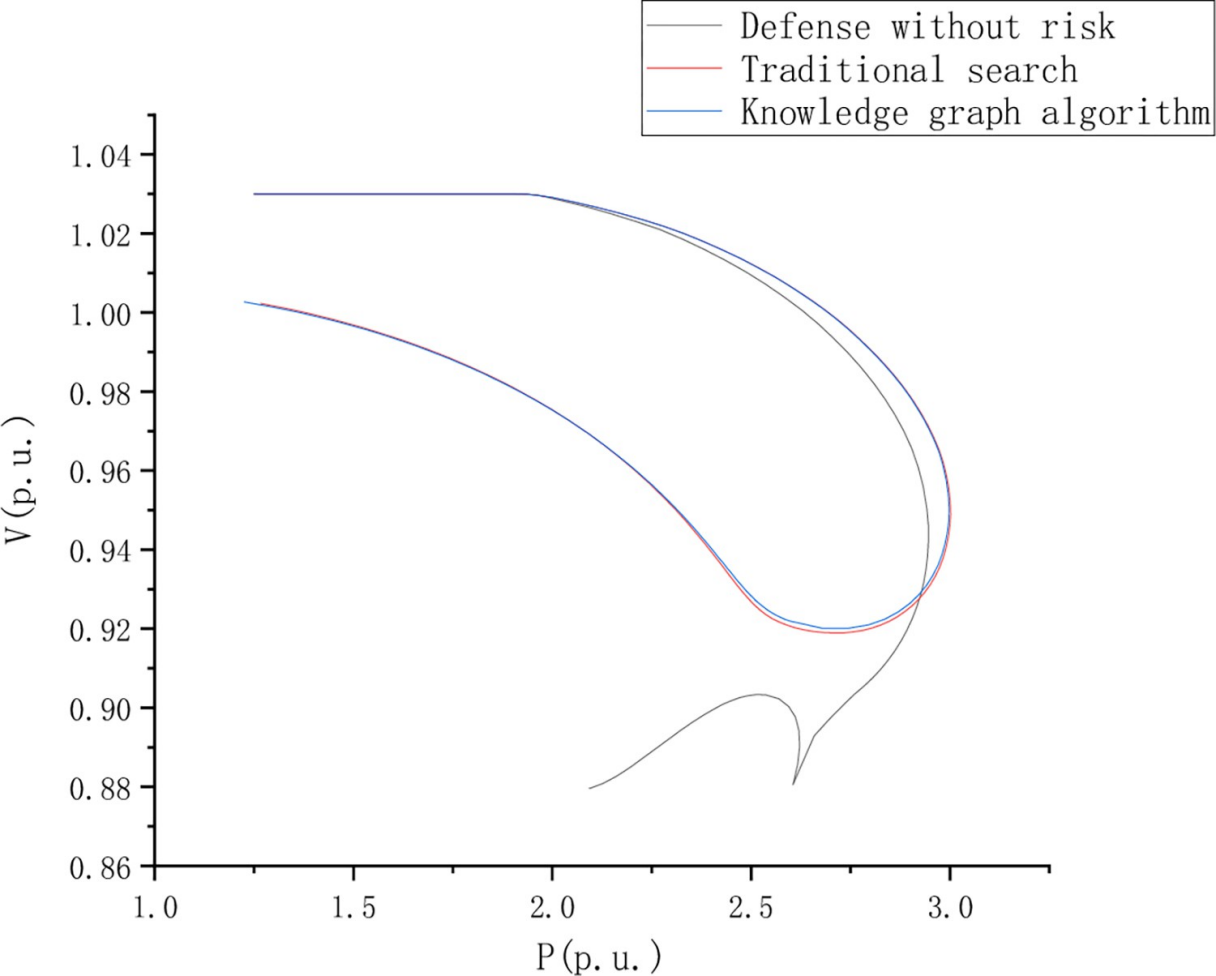
PV curve of point 44 when node 8 load deficiency period.

**Fig 8:**
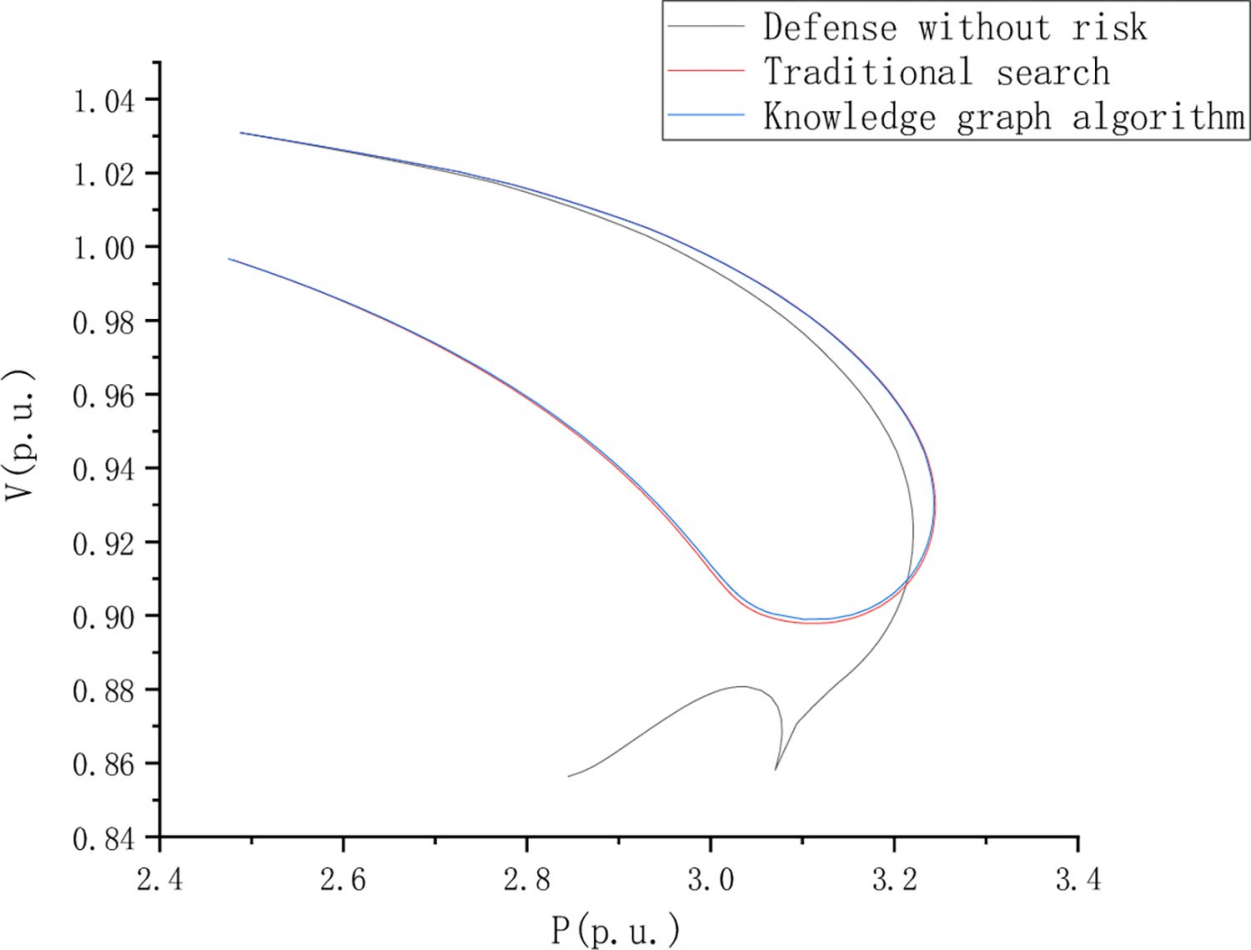
PV curve of node 13 with load shortage.

**Fig 9:**
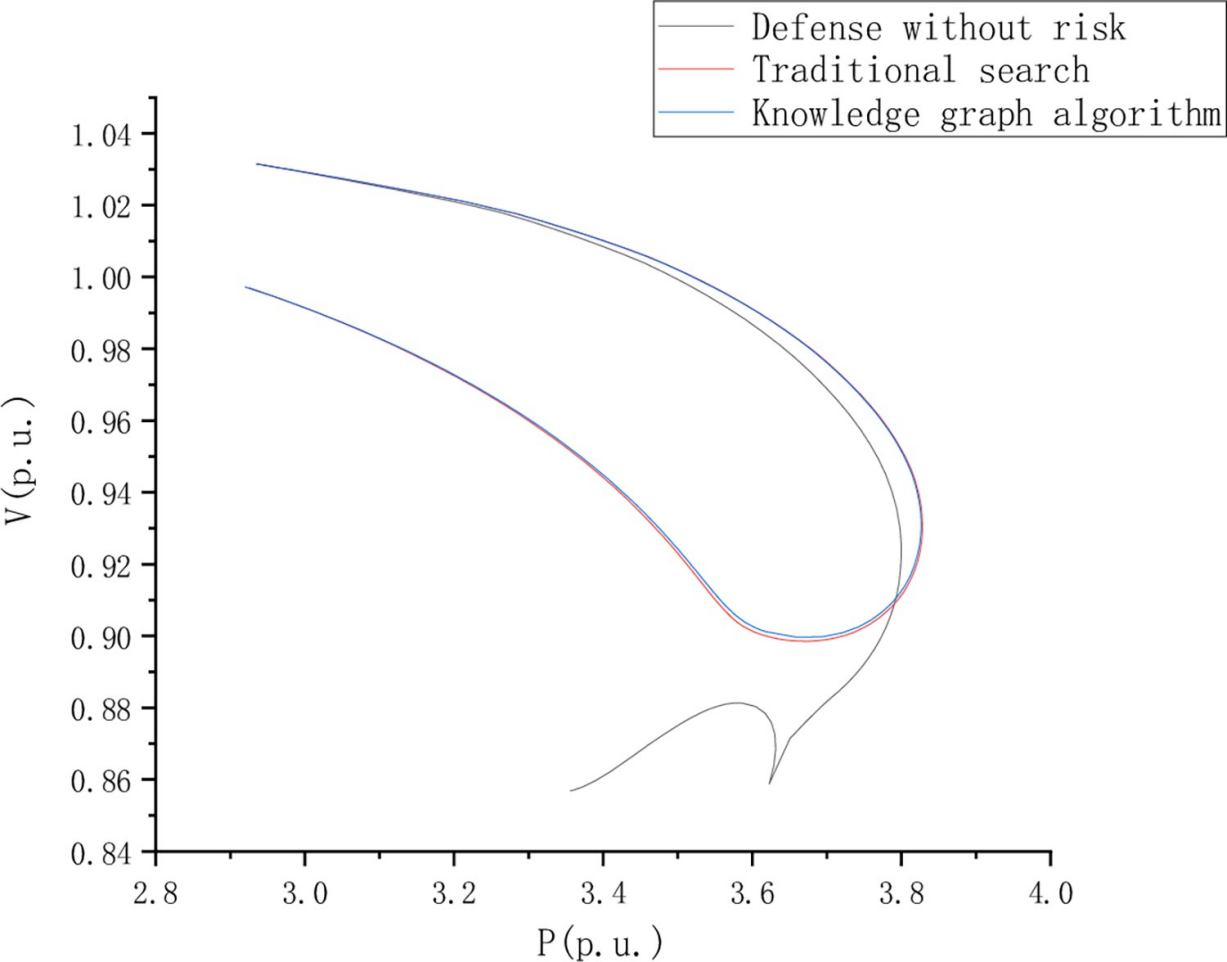
PV curve of point 44 during the load shortage period of node 13.

Distribution network fault detection test analysis, To set the control group, the PV curves of nodes 8, 13, 22, and 41 under dynamic load changes were analyzed respectively. This paper compares and analyzes the riskless defense, traditional search, and knowledge graph algorithms. Fig 6 shows the PV curve of node 8 when the load is short, and Fig 7 shows the PV curve of point 44 when the load is short, Figs 6–9 is the comparison of waveforms of different fault points in the knowledge graph, which is a quantitative analysis. The advantage of a knowledge graph is that it combines intelligent algorithms to record curves, which speeds up the subsequent analysis and recognition.

**Fig 10:**
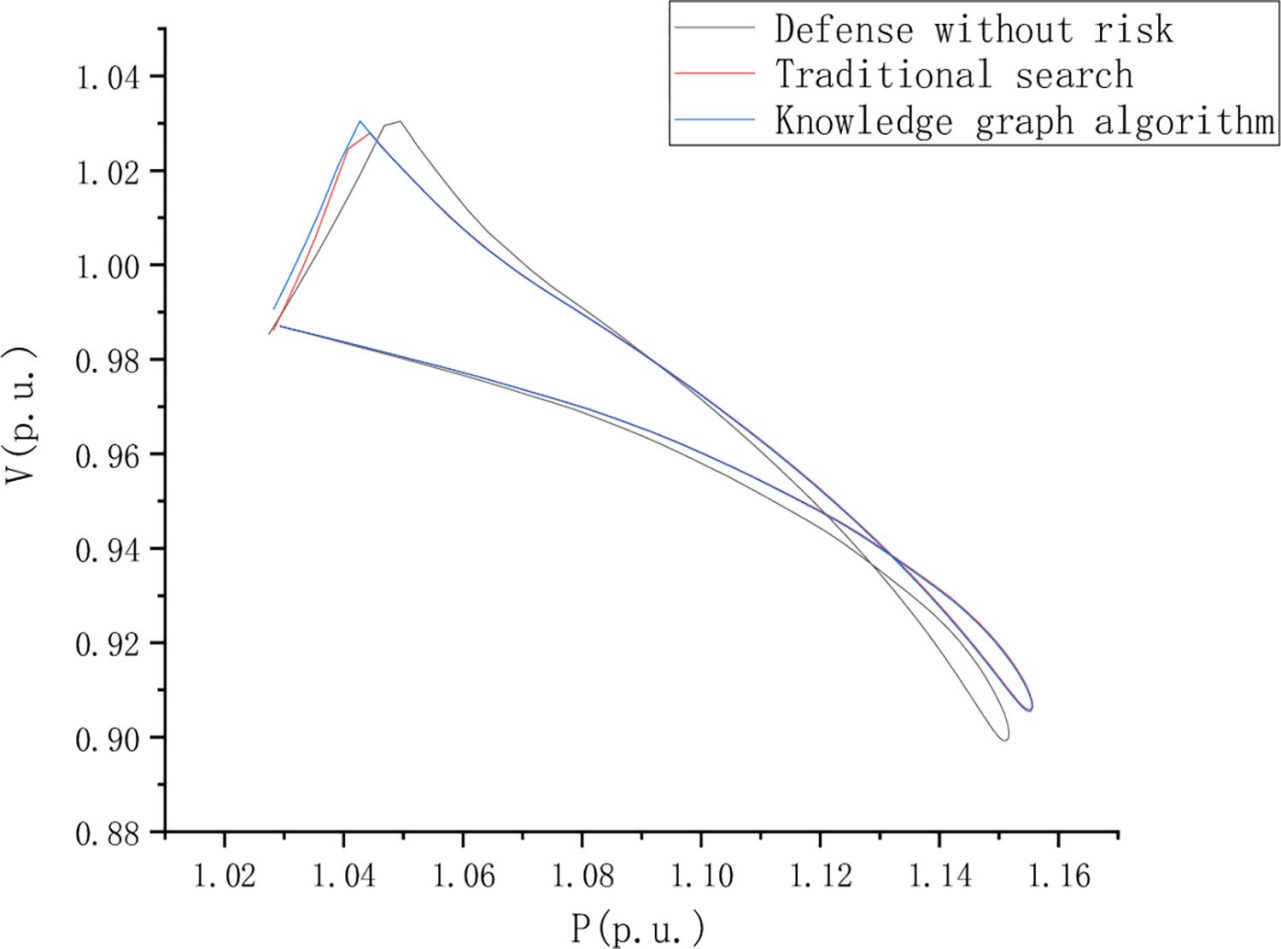
PV curve of point 44 during the load deficiency period of node 22.

PV curve reflects the AC voltage robustness in the journey and compares and analyzes the risk-free state defense, traditional search, and knowledge graph algorithms. However, the PV curve shows a better voltage stability margin in the return journey stage. Fig 8 is the PV curve of node 13 when the load is short, Fig 9 is the PV curve of point 44 when the load is short, and Fig 10 is the PV curve of point 44 when the load is short, of node 22.

**Fig 11:**
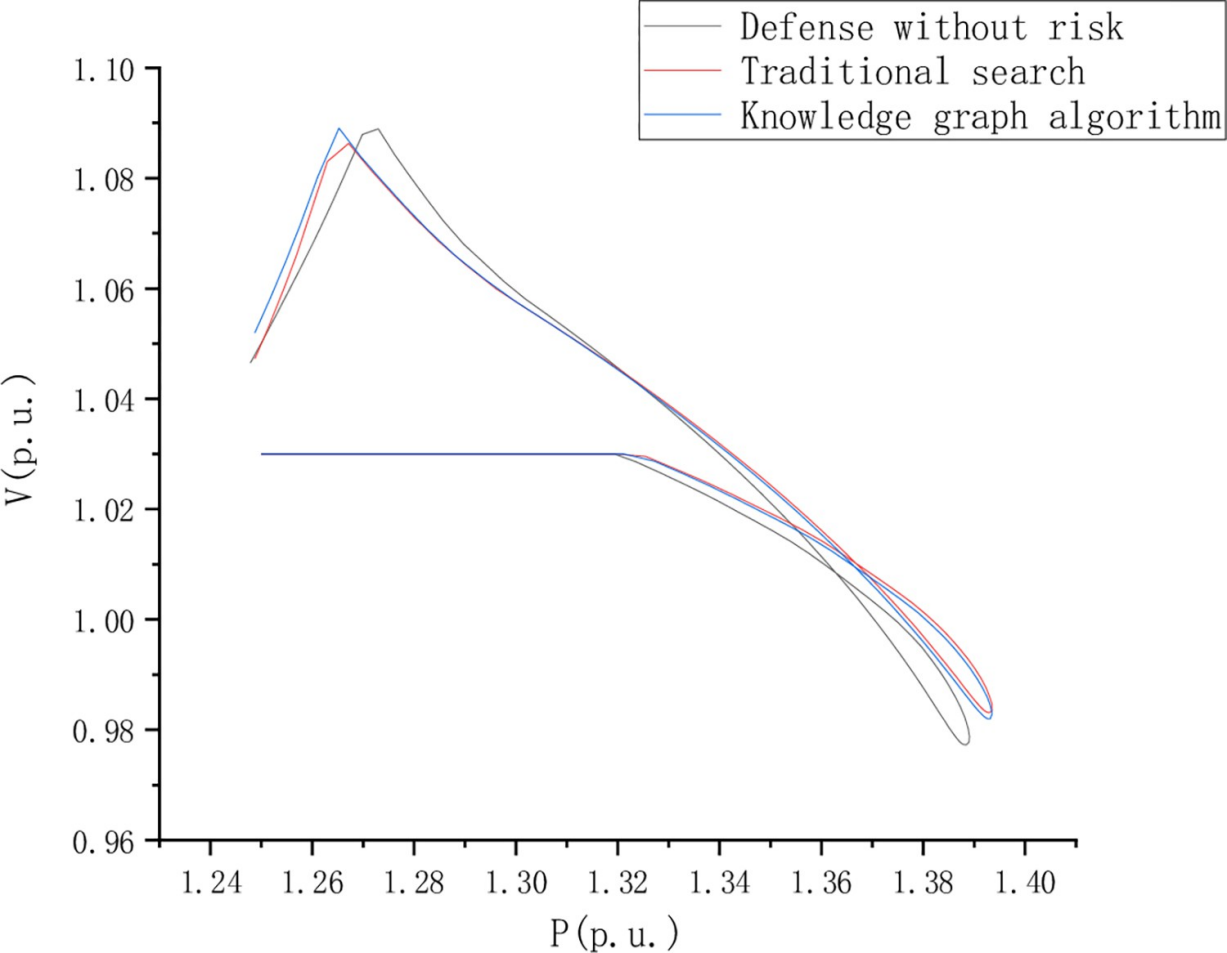
PV curve of point 44 during the load deficiency period of node 22.

For the PV curve of node 44, the voltage stability of the red curve is higher than that of the blue curve as per unit value of active power decreases. Fig 11 PV curve of point 44 during the load deficiency period of node 22.

In the case of the knowledge graph algorithm, with the increase of power, the voltage drop of the PV curve in the outbound process is controlled, but the voltage drop of the PV curve in the return stage is more obvious. However, compared with the case of adjusting the camera on the AC side of the DC near area, the voltage stability margin is significantly improved.

**Fig 12:**
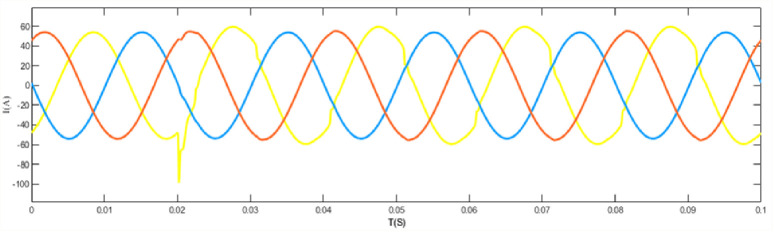
Ungrounded system node 44 arc grounding fault model and fault waveform of the neutral point of the grid structure waveform.

**Fig 13:**
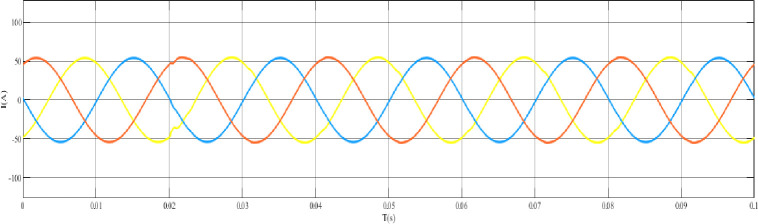
Failure waveform of node 44 in the ungrounded system with a neutral point of the grid structure Node 8 when arc grounding fault occurs.

**Fig 14:**
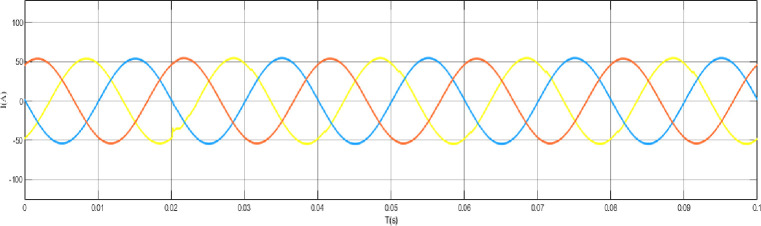
Fault waveform of node 44 in the ungrounded system with a neutral point of the grid structure Node 13 when arc grounding fault occurs.

**Fig 15:**
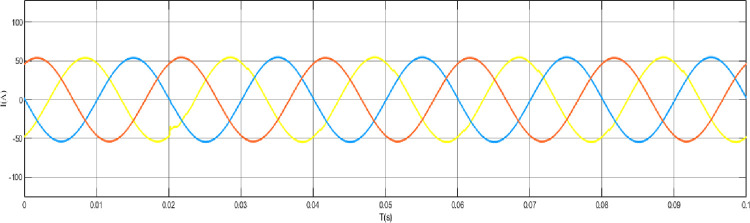
Ungrounded system node 44 fault waveform of node 22 when arc grounding fault occurs in the neutral point of the network structure.

**Fig 16:**
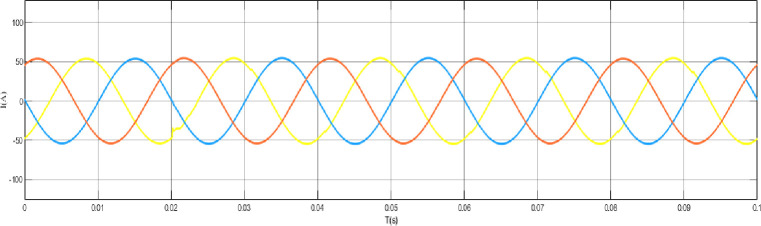
Fault waveform of node 44 in the ungrounded system with a neutral point of the grid structure node 41 when arc grounding fault occurs.

When the grid structure is not grounded at the neutral point, the load is set as a balanced load, the line analog capacitance is 0.44uF, and an arc grounding fault occurs at node 44. The output curve is shown in the Fig 12 arc grounding fault model and fault waveform of neutral point ungrounded system node 44, Fig 13 arc grounding fault waveform of neutral point ungrounded system node 44 at the time of arc grounding fault, and Fig 14 arc grounding fault waveform of node 13 at the time of arc grounding fault of neutral point ungrounded system node 44. Fig 15 Network structure neutral point ungrounded system node 44 arc grounding fault node 22 fault waveform Fig 16 Network structure neutral point ungrounded system node 44 arc grounding fault node 41 fault waveform.

## 6. Conclusions

In this paper, the knowledge graph model of distribution network risk analysis is constructed, and the multi-dimensional distribution network fault disposal and knowledge graph construction oriented to feeder and station area is designed, Considering the distribution line parameters, neutral grounding mode, and different fault types of the low-voltage distribution network model, the search for a non-planned island based on the knowledge graph adjacency matrix is studied.

Combined with simulation experiments, it is verified that the proposed method can effectively depict the information risk process of the distribution network. It is verified that the proposed method can effectively describe the information risk process of the distribution network.
