# Correction: Research on distribution network fault processing technology based on knowledge of graph

**DOI:** 10.1371/journal.pone.0327292

**Published:** 2025-06-27

**Authors:** Qiang Li, Feng Zhao, Li Zhuang, Jiangwen Su, Xiaodong Zhang

The Funding statement for this article is incorrect. The correct Funding statement is as follows: Supported by the two-level collaborative R&D project of State Grid Information and Communication Industry Group, “Research and Application of Key Technologies for Building a National Artificial Intelligence Open Innovation Platform for New Power System”. The number is YR-DLL-BJ-RJKF-221014–03219.
